# *Caspase 8* gene expression highly reduced in atherosclerotic model cells: effect of oxidized low-density lipoprotein

**DOI:** 10.7717/peerj.21671

**Published:** 2026-09-07

**Authors:** Burcu Bayyurt, Lutfi Bayyurt

**Affiliations:** 1 Department of Medical Biology, Sivas Cumhuriyet University, Sivas, Turkey; 2 Department of Animal Science, Tokat Gaziosmanpaşa University, Tokat, Turkey

**Keywords:** *Caspase 8*, Gene expression, Human umbilical vein endothelial cell, Oxidized low-density lipoprotein

## Abstract

**Background and Aim:**

Apoptosis is one of the programmed cell death pathways. Cysteine protease, Caspase 8 (*CASP8*), starts apoptosis signaling through the extrinsic apoptotic pathway. Oxidatively modified low-density lipoprotein (ox-LDL) activates the apoptosis in vascular endothelial cells (VECs). Ox-LDL is a crucial risk factor that ultimately promoted development of atherosclerosis (AS). We purposed to investigated gene expression of *CASP8* in human umbilical vein endothelial cells (HUVECs) after ox-LDL dose-dependent stimulation.

**Methods:**

We examined *CASP8* gene expression level in treated two different dose ox-LDL treated HUVECs and HUVECs without ox-LDL in current research. We made measurement with dye of Symmetrical Cyanine Benzothiazole Green (SYBR Green) utilizing the quantitative real-time polymerase chain reaction (qPCR) method.

**Results:**

When we compared to HUVECs without treatment of ox-LDL, expression of *CASP8* was statistically significant down-regulated (*P* < 0.05) in HUVECs stimulated with ox-LDL. In addition, we supported our gene expression results with permutation analysis of variance (ANOVA).

**Conclusion:**

Ox-LDL may affect expression of *CASP8* therefore apoptosis signaling pathway in VECs. Excessive down-regulation in gene expression of *CASP8* in HUVECs stimulated by ox-LDL may show potential role of it in the endothelial cell (EC) apoptosis for AS.

## Introduction

Apoptosis is a common cell death form and an effective process in many pathological conditions including cardiovascular diseases (CVDs). Apoptosis plays an important role in the development of CVDs, particularly in atherosclerosis (AS). Endothelial cell apoptosis is considered an early event in AS and contributes to endothelial dysfunction and plaque development. Increased apoptosis of vascular cells may impair endothelial integrity and promote atherosclerotic progression. Therefore, dysregulation of apoptotic pathways is closely associated with the pathogenesis of CVDs (Ni et al., 2024; Kanavos & Birbas, 2025; Wang et al., 2026a; Wang et al., 2026b). The apoptosis initiation and regulation is highly controlled by a proteolytic enzymes family, the caspases. As initiator caspase, Caspase 8 (CASP8), is involved in the extrinsic apoptosis pathway for answering to death receptors ligations (Zhong et al., 2020). External stimuli binds to diverse death receptors of cell transmembrane and activates extrinsic apoptotic pathway. After receptor activation, various intracellular proteins influence each other to lead a signaling complex of death-inducing. The complexes prompt downstream initiator caspases, such as *CASP8*. *CASP8* can activate intrinsic apoptotic pathway *via* stimulating mitochondrial stress, or can induce downstream effector caspases, which directly initiate cell components degradation and facilitate death of cell. When progressing during the apoptosis phases, *CASP8* interacts with many apoptotic proteins in intracellular chemical signaling pathway (Goldblatt, Cirka & Billiar, 2021). Vascular endothelial cells plays critical role in sustaining homeostasis. Endothelial cells are active metabolic biological tissue components that has numerous crucial physiological functions (Lawal, Davids & Marnewick, 2016). It is commonly accepted that injury of endothelial cell (EC) acts as important player in diverse human diseases such as AS (Kang et al., 2021). Endothelial cell injury is considered to be an early in development of AS for CVDs (Monteiro et al., 2019). Atherosclerosis is the main chronic inflammatorydisease which is the primary cause of CVDs (Gao et al., 2022). It is characterized by artery intima thickening (Zhao et al., 2022) and associated with lipid metabolism disorders owing to possibly underlying inflammation (Wang et al., 2023). Atherosclerosis pathogenesis is unclear. LDL is oxidized in arterial wall subendothelial space for becoming ox-LDL that causes vascular endothelial injury. Oxidatively modified low-density lipoprotein (ox-LDL) further exacerbates the AS (Guo et al., 2022). Apoptosis plays a critical role in endothelial dysfunction and the development of CVDs. EC injury is a key event in the initation of the AS and ox-LDL is one of the major factors that induce endothelial dysfunction and cell death (Tian et al., 2026). Caspases, a family of cysteine-dependent aspartate proteases, play a central role in the regulation of apoptosis in CVDs, and multiple atherosclerotic risk factors are known to induce apoptotic pathways (Gundapaneni et al., 2017). *CASP8* is a key regulator of cell death pathways, including apoptosis, necroptosis, and inflammation, and therefore plays an important role in vascular pathology (Zhang et al., 2024). Previous studies have demonstrated that altered expression of *CASP8* is associated with CVDs and may be involved in disease progression (Zhang et al., 2024; Pavić et al., 2024). Gene expression analyses in cardiovascular patient cohorts have shown significant alterations in *CASP8* expression, suggesting that dysregulation of apoptotic pathways contributes to cardiovascular pathology (Pavić et al., 2024). Ox-LDL induces apoptosis in VECs (Li & Mehta, 2000; Chen et al., 2004) and vascular smooth muscle cells (Nishio, Arimura & Watanabe, 1996). It has shown that caspase-8 play roles in the ox-LDL-induced apoptotic signaling pathway (Chen et al., 2004) in human coronary artery endothelial cells. Although ox-LDL-induced endothelial apoptosis has been widely reported, the direct effect of ox-LDL on *CASP8* gene expression in ECs has not been clearly elucidated. Therefore, in this study, we purposed to investigate the effect of ox-LDL on *CASP8* gene expression in human umbilical vein endothelial cells (HUVECs). Investigating the effect of ox-LDL on *CASP8* gene expression may provide important insights into the molecular mechanisms of endothelial injury and development of CVDs such as AS. Since *CASP8* is a key regulator of apoptosis and its altered expression has been associated with CVDs, we hypothesized that ox-LDL exposure may modulate *CASP8* gene expression in ECs and therefore, contribute to development of AS. Thus, we aimed to search that whether ox-LDL alter expression of *CASP8* gene HUVECs. *CASP8* gene expression was compared between HUVECs stimulated with ox-LDL and HUVECs without ox-LDL stimulation.

## Materials and Methods

### Human umbilical vein endothelial cells and ox-LDL treatments

Human umbilical vein endothelial cells were ensured from American Type Culture Collection. HUVECs were grown in Dulbecco’s Modified Eagle Medium (DMEM, CAPRICORN (Capricorn Scientific GmbH)) supplemented with 1% penicillin-streptomycin (Capricorn Scientific GmbH) and 10% fetal bovine serum (Capricorn Scientific GmbH) at 37 °C, 5% CO2 and under 95% humidity. Every 2–3 days, the HUVECs were picked up from the culture medium using 0.25% trypsin and passaged. The cells in the logarithmic growth phase, at ∼80–90% confluence, were utilized for experiments (Liu et al., 2020). HUVECs were categorized into two groups: Experiment groups in which HUVECs were grown in supplemented DMEM with 25 (Group 1) and 40 µg/ml ox-LDL concentration (Group 2) (Invitrogen LOT2160046, L34357 (Thermo Fisher Scientific, Waltham, MA, USA)) for 24 h (Gong et al., 2019). The other group is control group in which HUVECs were grown in supplemented DMEM without ox-LDL. Two different ox-LDL concentrations (25 µg/mL and 40 µg/mL) were used to evaluate whether ox-LDL has a dose-dependent effect on *CASP8* gene expression in ECs. These concentrations were selected based on previous studies in the literature in which similar ox-LDL concentrations were used to induce endothelial dysfunction and cell injury without causing excessive cell death (Zhang & Jiang, 2016; Gong et al., 2019; Ma et al., 2024). Ox-LDL concentration level at which cell viability began to be affected was 25 µg/ml (Ma et al., 2024). Cell density decreased significantly after the application of 40 µg/ml ox-LDL in HUVECs and experiments were carried out with these two ox-LDL concentrations. Same consumables were used across all experimental conditions. In addition, freshly thawed cells were used in experiments.

### Gene expression

RNeasy Mini Kit (Qiagen, Hilden, Germany) (QIAGEN, catalog no: 74104) was provided to extracted total RNA from the HUVECs. Complementary DNA was synthesized by utilized kit of reverse transcription (A.B.T.™ with RNase Inh. High Capacity, catalog no: C03-01-20) (A.B.T, Ankara, Turkey). Quantitative real-time polymerase chain reaction (qPCR) was performed using Symmetrical Cyanine Benzothiazole Green (SYBR Green) master mix (A.B.T.™ 2X qPCR SYBR-Green MasterMix kit, catalog no: Q03-02-05) (A.B.T, Ankara) on LightCycler 96 (Roche, Switzerland). We provided primers of *CASP8* and *Glyceraldehyde 3-phosphate dehydrogenase* (*GAPDH*) genes as optimized assays in qPCR. Forward (forward primer serial no: 20220609/1-115, 5′GATGTTATTCCAGAGACTCCAG 3′) and reverse primer (reverse primer serial no: 20220609/1-116, 5′GGTAGGTAATCAGCAAATCCA 3′) specific to human *CASP8* was used for qPCR. *GAPDH* (forward primer serial no: 20201207-99, 5′ACAACTTTGGTATCGTGGAAGG 3′; reverse primer serial no: 20201207-100, 5′GCCATCACGCCACAGTTTC 3′) was housekeeping (internal control) gene utilized in qPCR (Table 1). QPCR were performed in three replicates. No-template controls was included in the qPCR experiments, and melting curve analysis was performed to confirm the specificity of the qPCR products.

**Table 1:** Primer sequences used for qPCR analysis.

Gene	Primer sequence (5′- 3′)	Product length (base pair)
*GAPDH*	Forward: ACAACTTTGGTATCGTGGAAGG	101
	Reverse: GCCATCACGCCACAGTTTC	
*CASP8*	Forward: GATGTTATTCCAGAGACTCCAG	
	Reverse: GGTAGGTAATCAGCAAATCCA	110

**Notes.**

*CASP8*, *Caspase 8*; *GAPDH*, *lyceraldehyde 3-phosphate dehydrogenase*, *CASP8* was used as the target gene and GAPDH was used as the reference gene.

### Statistic analysis

We uploaded threshold cycle (Cq, Ct) values to “GeneGlobe Data Analysis Center” (https://geneglobe.qiagen.com/us/analyze; QIAGEN, Hilden, Germany) to analyze data of the *CASP8* gene expression. Gene expression ratios (fold change (FC) calculations) were calculated according to the 2^−ΔΔCT^ method. FC was calculated as ratio of the relative gene expression between the control and experiment groups (Group 1 and Group 2). Values higher than 1 show up-regulation (increased gene expression), while values between 0 and 1 show down-regulation (decreased gene expression). FC values equal to 1 shows no change between the groups. If a FC value greater than 1, the fold regulation (FR) and FC values are the same. For FC values less than 1, the FR is the negative inverse of the fold change value. Mann–Whitney U test was performed using delta Ct (ΔCt) values (CASP8_Ct_-GAPDH_Ct_ in all groups) to calculate *P* value. ΔCt values are normalized values. *P* values lower than 0.05 were accepted statistically significant (Livak & Schmittgen, 2001).

In this study, because the ΔCt values for the *CASP8* gene did not contain within-group variance in the ox-LDL-treated groups, estimation of the error term (within-group variance), one of the fundamental assumptions of one-way analysis of variance (ANOVA), was not achieved. Therefore, classical parametric one-way ANOVA is not statistically applicable. To reliably test group effects without adhering to parametric assumptions, a permutation-based one-way analysis of variance with 5,000 replicates was preferred for *CASP8* gene. Permutation ANOVA provides a more robust and assumption-free testing approach under conditions of zero variance and small samples because it allows the evaluation of observed group differences based on the empirical distribution generated by random resampling. Effect size analyses were performed to assess the magnitude of the difference between the dose groups and the control group. Because repeated measurements at the 25 and 40 µg/ml ox-LDL doses did not reveal any variance, the classical Cohen’s d coefficient could not be calculated; instead, the Glass Δ effect size, based on the standard deviation of the control group, was used. High Glass Δ values were interpreted to indicate the magnitude of the biological effect of the ox-LDL dose on *CASP8* gene expression. All analyses were conducted in Python (v3.12) using the SciPy and NumPy packages.

### External transcriptomic validation using the GSE137578 dataset

The public transcriptomic dataset GSE137578 from the Gene Expression Omnibus (GEO) database (https://www.ncbi.nlm.nih.gov/geo/query/acc.cgi?acc=GSE:GSE137578) was used to externally validate our qPCR findings. This dataset contains gene expression profiles of human aortic endothelial cells (HAECs) exposed to ox-LDL and untreated control cells. Differential expression analysis between ox-LDL-treated and control samples was performed using the limma package in R. Expression data were normalized according to the original GEO platform processing procedures, and empirical Bayes moderated t-tests were applied to identify differentially expressed genes. *P*-values were adjusted for multiple testing using the Benjamini–Hochberg false discovery rate (FDR) method. To specifically evaluate the consistency of the experimental findings, expression change of *CASP8* was extracted from the differential expression results. The expression distribution of *CASP8* was additionally visualized using boxplots displaying individual biological replicates and mean ± standard deviation values.

## Results

**Figure 1:**
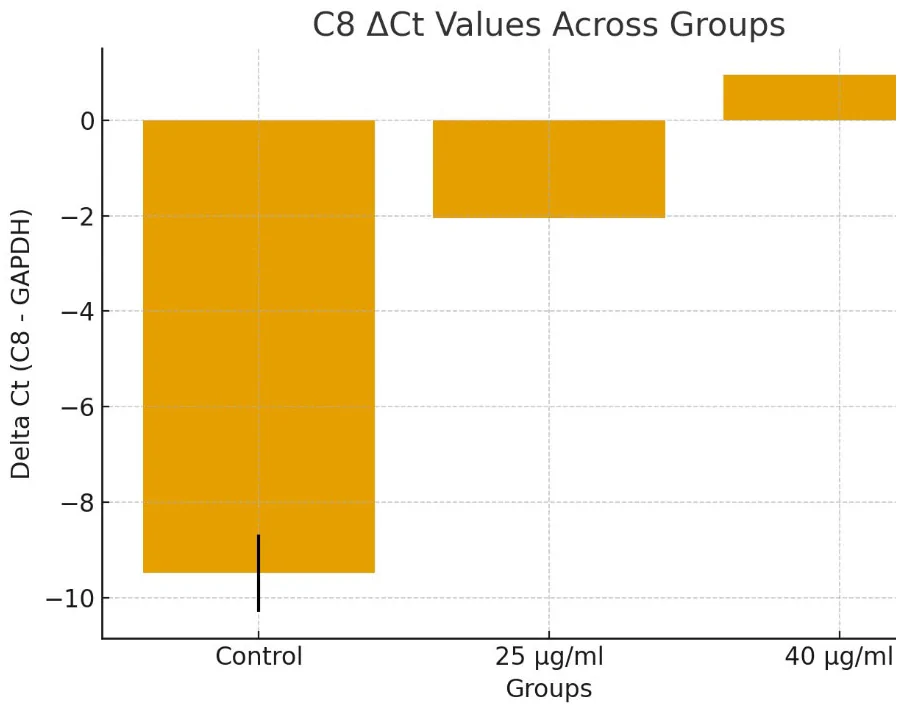
*CASP8*Δ Ct values in control and ox-LDL-treated HUVECs. HUVECs were treated with 25 µg/mL (Group 1) and 40 µg/mL (Group 2) ox-LDL and compared with untreated control cells (Control). Gene expression levels were calculated using the Δ Ct method (Ct CASP8 –Ct GAPDH). Each group included three technical replicates. Statistical comparisons between groups were performed using the Mann–Whitney U test due to the small sample size. *CASP8* gene expression was significantly down-regulated in both Group 1 and Group 2 compared to the control group (*P* = 0.03). Furthermore, *CASP8* expression was significantly lower in Group 2 than in Group 1 (*P* < 0.001). Lower ΔCt values indicate higher gene expression.

We measured *CASP8* gene expression level in experiment groups compared to the control group after qPCR. Ct values of gene of interest (*CASP8*) and housekeeping gene (*GAPDH*) in groups were utilized for calculating difference in gene expression. For *CASP8* gene, mean of the Ct values was 26.49 and 28.34 in Group 1 and Group 2, respectively while control group’ mean of Ct was 18.73. For *GAPDH* gene, mean of the Ct values in Group 1, Group 2 and control groups were 28.54, 27.39 and 28.21, respectively. We compared *CASP8* gene expression level between HUVEC stimulated with 25 µg/ml ox-LDL and 40 µg/ml ox-LDL (experiment groups) and HUVEC with no ox-LDL stimulation (control group). Delta Ct values representing the relative expression level of the *CASP8* gene compared to the reference gene in the control and experimental groups were demonstrated in Fig. 1. An examination of the ΔCt values for the *CASP8* gene clearly demonstrates that ox-LDL administration exerts a highly potent suppressive effect on gene expression, increasing with dose. In the control group, the ΔCt value was approximately −9, but in the 25 µg/ml ox-LDL group, it increased to −2. In 40 µg/ml ox-LDL group, this value was higher than 0 (Fig. 1). This significant increase demonstrated a significant decrease in *CASP8* gene expression compared to the control group. The fact that ΔCt reached approximately 1 in the highest dose group, the 40 µg/ml group, indicating a relative down-regulation of *CASP8* gene expression. Repeated measurements yielded identical values in the 25 and 40 µg/ml groups, in particular, supports the strong and consistent dose response. Overall, the graph clearly demonstrates a significant and dose-related down-regulation of the *CASP8* gene against ox-LDL (Fig. 1). Expression of the *CASP8* gene was down-regulated statistically significant in Group 1 and Group 2 when compared to control group (*P* = 0.03). *CASP8* expression was also compared between Group 1 and Group 2. *CASP8* was down-regulated statistically significant in Group 2 compared Group 1 (*P* < 0.001). Fold change and FR values are in Table 2.

**Table 2:** Fold change of *CASP8* gene expression between groups.

**Gene**		**Fold change (comparing to control)**		
	**Group 1**		**Group 2**	
	Fold change Fold regulation	*P* value	Fold change Fold regulation	*P* value
*GAPDH*	1.00	Nan	1.00	Nan
*CASP8*	0.01–172.84	0.03^*^	0.00–1,382.76	0.03^*^
		**Group 1 comparing to Group 2**		
*GAPDH*	1.00	Nan		
*CASP8*	0.12–8.00	<0.001^*^		

**Notes.**

*CASP8* *Caspase 8* *GAPDH* *Glyceraldehyde 3-phosphate dehydrogenase* *P* value Significant value Ct Threshold cycle * Statistic significant Group 1 HUVECs were grown in supplemented DMEM with 25 µg/ml ox-LDL Group 2 HUVECs were grown in supplemented DMEM with 40 µg/ml ox-LDL - sign indicates down-regulation Nan Not a number

indicates undefined numerical value for reference gene *GAPDH*, therefore does not have a fold regulation value for *GAPDH*.

Variance analyses performed on ΔCt values for the *CASP8* gene showed statistically significant differences between the dose groups. Parametric one-way ANOVA revealed a statistically significant difference for *CASP8* with *F* = 400.69 and *p* = 4.1 ×10^−7^. Due to the small sample size and low within-group variance, an ANOVA with 5,000 permutations yielded similar statistically significant results (p_perm = 0.0024). The same F value in both tests indicates that the observed difference is strong and consistent, and the preservation of significance in the permutation test indicates that the results are robust, regardless of distributional assumptions (Table 3). Glass Δ values indicate that both 25 µg/ml and 40 µg/ml ox-LDL doses have a highly potent suppressive effect on *CASP8* gene expression. The effect size of 9.23 obtained in the comparison of control and HUVEC treated with 25 µg/ml ox-LDL indicates a significant reduction in *CASP8* gene expression. The very high Glass Δ value of 12.96 in the comparison of control and HUVEC treated with 40 µg/ml ox-LDL indicates that the high dose of ox-LDL almost completely silences the *CASP8* gene (Table 4).

**Table 3:** Permanova analysis results based on ΔCt for the *CASP8* gene.

**Test type**	***F* value**	***P*-value**
**Permütasyon ANOVA (5,000 perm.)**	400.6944	0.0024^*^

**Notes.**

*P*-value, Significant value.

* Statistic significant, ΔCt: Delta Ct, *CASP8*: *Caspase 8*.

**Table 4:** Effect sizes in the *CASP8* gene (GlassΔ).

**Groups**	**Average difference (ΔCt)**	**SD (Control)**	**Glass Δ**
**Control *vs* 25 µg/ml**	7.43	0.805	9.23
**Control *vs* 40 µg/ml**	10.43	0.805	12.96

**Notes.**

ΔCt Delta Ct SD Standard deviation *CASP8* *Caspase 8*

### External transcriptomic validation of *CASP8* expression using the GSE137578 dataset

**Figure 2:**
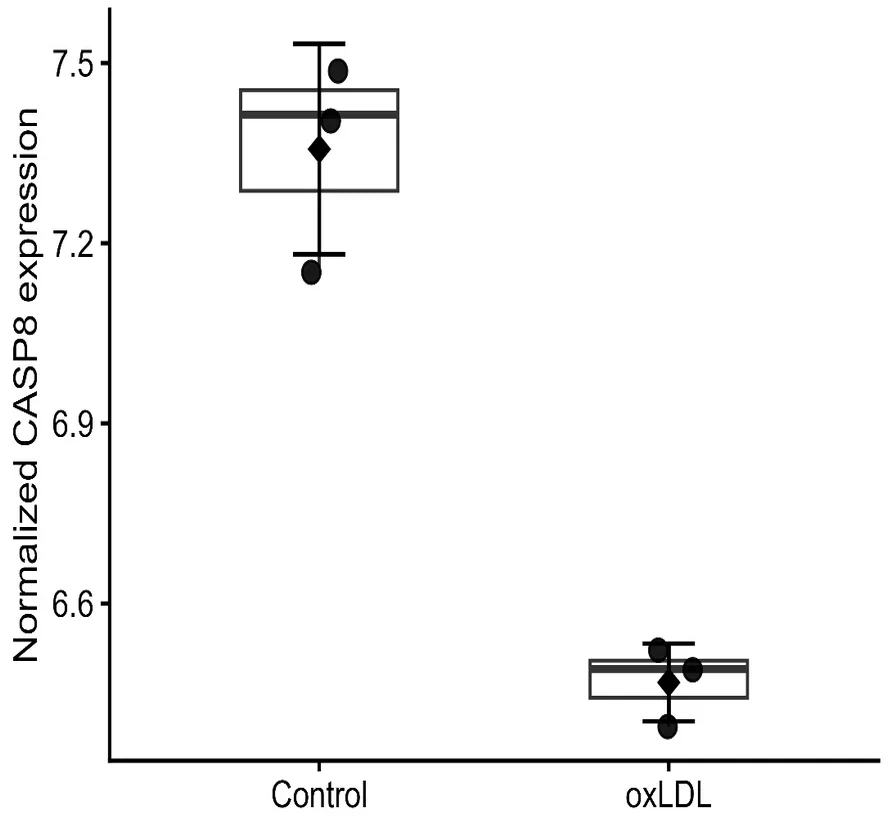
External transcriptomic validation of *CASP8* expression using the independent GSE137578 dataset. Expression levels of *CASP8* in untreated control and ox-LDL-treated human aortic endothelial cells (HAECs) obtained from the independent GSE137578 dataset. Individual points represent biological replicates (*n* = 3 per group). Boxes indicate the interquartile range, horizontal lines represent the median, and error bars show the mean ±standard deviation. Differential expression analysis demonstrated significant down-regulation of *CASP8* in ox-LDL-treated endothelial cells (ECs) compared with untreated controls (log2FC = −0.888, adjusted *P* = 0.0035). This expression pattern was consistent with our qPCR findings obtained in the present study.

Independent public transcriptomic dataset GSE137578 was analyzed to externally validate the qPCR findings. This dataset contains HAECs exposed to ox-LDL and untreated control cells. Differential expression analysis identified significant down-regulation of *CASP8* in ox-LDL-treated ECs compared with controls (log2FC = −0.888, adjusted *P* = 0.0035; Table 5). The expression distribution of *CASP8* across control and ox-LDL-treated HAEC samples is presented in Fig. 2. Consistent with the qPCR results obtained in the present study, *CASP8* expression was reduced in the ox-LDL-treated group. This provides independent transcriptomic support for the observed down-regulation of *CASP8* after ox-LDL stimulation.

**Table 5:** Differential expression results of *CASP8* in the independent GSE137578 validation dataset (ox-LDL-treated HAECs *versus* untreated controls).

**Gene**	**Probe ID**	**log2FC**	***P*-value**	**Adjusted *P*-value (FDR)**	**Regulation**
*CASP8*	A_24_P157087	−0.888	0.00035	0.00354	Down-regulated

**Notes.**

Differential expression analysis was performed using the limma package. Positive log2FC values indicate higher expression in ox-LDL-treated cells, whereas negative log2FC values indicate lower expression compared with untreated controls. Statistically significant genes after Benjamini–Hochberg correction (FDR < 0.05) were *CASP8, CASP8*, *Caspase 8*; HAECs, Human aortic endothelial cells.

## Discussion

Suitable regulation of cell death is serious, since both reduced and excessive apoptotic rates can cause to initiation of CVDs. Though early AS mechanisms are still a research effective zone, it is known that the layer of VEC which lines artery wall is disrupted in the AS. The layer of endothel continuously proliferates for renewing the intimal layer of blood vessels. Vascular endothelial cells expose to the apoptosis result from overcrowding or injury (Ohsawa, Vaughen & Igaki, 2018).

Caspase 8 is central factor in pathways of cell death (Kesavardhana, Malireddi & Kanneganti, 2020; Orning & Lien, 2021). It was implicated that the observed activation of *CASP8* was accompanied by the presence of main apoptosis hallmarks (Cristaldi et al., 2023). Dysregulation in expression levels or activity is associated with the numerous human diseases pathogenesis, such as CVDs included in AS. Studies show that caspase activity modulation can crucially ameliorate progression of the disease (Zhang et al., 2024).

We focus on how ox-LDL may effect apoptosis in terms of *CASP8* gene expression in the cardiovascular system and therefore contribute to progression of CVDs. Thus, we compared *CASP8* gene expression of HUVECs in terms of ox-LDL exposure. Since HUVEC is among cell lines used for studying CVDs *in vitro*, we performed our experiment in HUVECs. In vitro models permit the study molecular and cellular mechanisms of the disease and supply a search platform to screen potential therapeutic agents. Although there are alternative models, like *ex vivo* models and animal models, *in vitro* models show higher experimental reproducibility and flexibility. *In vitro* models are a valuable tool to understand CVDs and thus AS pathophysiological mechanisms. Many risk agents of CVDs promote the expression of several apoptotic agents in ECs. Conclusively, activation of endothel can cause to dysfunction of the EC (Grego et al., 2025). Researches have demonstrated that injury of endothel is involved in pathogenesis of the AS, which involves the high EC apoptosis frequency in AS initial stages. Function and integrity of the endothelial is easily changed by inducing AS (Chen et al., 2004). Thus, the VEC apoptosis role in AS has attracted attention. A lot of studies have demonstrated that ECs are highly sensitive to lipid peroxidation and free radicals. Moreover, in an effort to clear the lipids, ECs change the lipid to foam cells which is major endothelial injury caused by ox-LDL (Wu et al., 2012).

Oxidized-LDL can activate apoptosis in a various tissues and cells including smooth muscle cells, coronary artery ECs, and macrophages (Chen et al., 2004). Several studies have proposed that ox-LDL has a serious role in the AS pathogenesis and the atherosclerotic plaque destabilization, and acute myocardial infarction (Chen et al., 2004). Oxidized-LDL is accepted risk factor to stimulate injury of the EC for contributing to the AS process under pathological conditions (Su et al., 2018; Li, Chen & Shi, 2022; Gao et al., 2024). Wu et al. (2012) explored *CASP8* protein expression involment to the pathway of ox-LDL-induced apoptosis of HUVECs (Wu et al., 2012). HUVECs stimulated with 80 mg/l ox-LDL for 24 h and *CASP8* activitiy was examined using western blot analysis. It was found that 80 mg/l ox-LDL-stimulated cells demonstrated an increased rate of apoptosis. In the study by Wu et al. (2012), HUVECs treated with 80 µg/mL ox-LDL for 24 h showed that no significant increase was observed in *CASP8* protein expression (Wu et al., 2012). In our study, HUVECs treated with 40 µg/mL ox-LDL showed a significant decrease in *CASP8* gene expression compared to both the control group and the 25 µg/mL ox-LDL group. These findings suggest that ox-LDL may differentially regulate caspase pathways depending on concentration and experimental conditions. While Wu et al. (2012) reported activation of the intrinsic apoptotic pathway, our findings indicate that *CASP8* expression may be suppressed under ox-LDL treatment, which may suggest alterations in the extrinsic apoptotic pathway. Ox-LDL induces transcriptomic and epigenomic changes in ECs (Jiang et al., 2025) and it reduces cell viability in a dose-dependent manner (Zhang & Jiang, 2016). Ox-LDL is known to has different biological effects depending on its concentration (Zhang & Jiang, 2016; Gong et al., 2019; Ma et al., 2024). Lower concentrations mainly induce endothelial dysfunction, whereas higher concentrations may induce apoptosis or other forms of cell death (He et al., 2025). In the present study, *CASP8* gene expression decreased with increasing ox-LDL concentrations. *CASP8* gene expression decreased in ox-LDL-treated groups compared to the control group, and the decrease was more pronounced in the 40 µg/mL ox-LDL group compared to the 25 µg/mL group. This finding suggests a dose-dependent effect of ox-LDL on *CASP8* gene expression in ECs. Previous studies have reported ox-LDL concentration-dependent effects on ECs (Wang et al., 2026a; Wang et al., 2026b). Therefore, our findings are consistent with the literature and suggest that increasing ox-LDL concentration may lead to greater suppression of *CASP8* expression in ECs. Previous gene expression study in patients with hypertrophic cardiomyopathy have reported dysregulation of apoptosis-related genes, including *CASP8*, and *CASP8* down-regulation has been reported in patient cohorts (Pavić et al., 2024). *CASP8* is not only an initiator of extrinsic apoptosis but also a key inhibitor (Oberst et al., 2011) and regulator of necroptosis and inflammation (Fritsch et al., 2019; Zhang et al., 2024). Therefore, decreased *CASP8* expression observed in our study may suggest a shift from apoptotic cell death toward alternative cell death pathways such as necroptosis under high ox-LDL exposure. This may be associated with endothelial dysfunction and inflammatory processes rather than classical apoptosis alone. These findings may help to explain the molecular mechanisms underlying endothelial dysfunction in CVDs including AS. However, further studies including protein-level and functional assays are required to clarify the exact cell death mechanism. The significant decrease in *CASP8* expression in HUVEC after ox-LDL treatment may result in apoptosis inhibition. This may accelerate the activation of an alternative cell death pathway such as necrosis and pyroptosis. It is clear that *CASP8* is a crucial determinant that effect and interplay among pyroptosis, apoptosis, and necroptosis, serving as a key regulator of cellular homeostasis and cell fate. *CASP8* functions as an important molecular regulator that inhibits necroptosis. When *CASP8* activity is reduced, receptor interacting serine/threonine kinase 1 and 3 form a necrosome complex that activates the necroptotic pathway, leading to inflammatory cell death (Zhang et al., 2024). Therefore, decreased expression of *CASP8* detected in our study may indicate suppression of apoptosis and activation of necroptosis-related pathways under ox-LDL exposure. In addition, in our previous study using the same experimental model and ox-LDL concentrations, *fas ligand* (FASL) gene expression was significantly higher in the 40 µg/mL ox-LDL group compared to the 25 µg/mL group (Bayyurt & Arslan, 2025). In the present study, however, *CASP8* expression was more strongly down-regulated at the higher ox-LDL concentration. Since FASL is an upstream activator of the extrinsic apoptotic pathway and *CASP8* is a downstream initiator caspase in this pathway (Zhang et al., 2024), these findings may indicate a dysregulation of the extrinsic apoptotic pathway under high ox-LDL conditions. The decrease in *CASP8* expression despite increased *FASL* expression may suggest impaired apoptotic signaling or a shift toward alternative regulated cell death pathways such as necroptosis. *CASP8* is known to function as a molecular switch among apoptosis, necroptosis, and pyroptosis (Fritsch et al., 2019). In this study, an independent transcriptomic validation analysis was also performed to validate a significant decrease in *CASP8* expression after ox-LDL treatment, and the GSE137578 dataset was examined. This dataset also showed a significant decrease in *CASP8* expression in HAECs treated with ox-LDL (Guo et al., 2024). The agreement between the qPCR results and the findings from the independent transcriptomic dataset suggests a reproducible endothelial response to ox-LDL stimulation. However, analysis of additional datasets of human atherosclerotic plaques revealed a different expression pattern. *CASP8* expression was found to be increased in atherosclerotic lesions compared to non-atherosclerotic arterial tissues in the GSE43292 and GSE100927 datasets. Higher *CASP8* expression was observed in advanced-stage plaques compared to early-stage plaques in the GSE28829 dataset (Meng et al., 2026). Transcriptomic studies performed on human carotid atherosclerotic tissues also show that significant changes occur in apoptosis-related pathways during inflammatory activation and plaque progression (Folkersen et al., 2012). Considering these findings together, it is understood that *CASP8* expression may vary depending on the different stages of AS. This interpretation is supported by studies showing that *CASP8* plays a significant role not only as an initiator of the extrinsic apoptotic pathway but also in the regulation of inflammatory signaling and immune cell activation (Boatright & Salvesen, 2003; Kearney et al., 2015). Furthermore, it is known that apoptotic and inflammatory signaling networks are closely related during atherosclerotic plaque development (Galluzzi et al., 2018). Considering all findings together, it is thought that *CASP8* may assume different roles in different stages of AS. Therefore, the differential expression patterns observed in human plaque tissues using EC models can be explained by differences in disease stage, cellular composition, and the local inflammatory environment. In the present study only *CASP8* gene expression was evaluated and performed in silico validation and comparisons. Further studies are planned to assess the expression and activation of key molecular markers of these pathways in order to better elucidate the underlying molecular mechanisms of different cell death. There are several limitations in the present study. First, apoptosis was evaluated only at the mRNA level through *CASP8* gene expression analysis. Additional apoptosis-related markers and protein level validation experiments were not included in this study. Therefore, our results should be interpreted as evidence of apoptosis-related gene expression changes rather than direct confirmation of apoptosis. Future studies including protein-level and functional apoptosis assays are needed to confirm these findings. Other limitations of this study are inability to test ox-LDL treatments in much different amounts for different durations and limited number of cell experiment replicates because of limited ox-LDL. Another limitation of this study is the lack of functional experiments to directly assess the role of *CASP8* in ox-LDL-treated ECs. Functional studies using *CASP8* knockdown or overexpression (*e.g.*, siRNA or CRISPR-Cas9 approaches) would help to clarify whether the observed changes in *CASP8* expression are directly related to ox-LDL.

## Conclusion

This paper examined impact of ox-LDL on *CASP8* gene expression. As a result, we have shown that ox-LDL decreased *CASP8* gene expression in HUVECs. Abnormal *CASP8* gene expression may be closely associated with AS prognosis. Our findings suggest that ox-LDL may effect apoptosis-related molecular changes in ECs through *CASP8*-associated pathways. In conclusion, the present study showed that ox-LDL treatment resulted in dose-dependent down-regulation of *CASP8* gene expression in ECs. These findings indicate that ox-LDL may influence apoptosis-related pathways at the transcriptional level. As this study was limited to gene expression analysis, and further studies including protein level and functional analyses are needed to better understand the role of *CASP8* in ox-LDL-induced EC injury.

## Supplemental Information

10.7717/peerj.21671/supp-1 Supplemental Information 1 qPCR raw data of all groups in HUVECs Each data indicates the value of threshold cycle (Ct or Cq) in all of groups (ox-LDL treatments and controls) in qPCR

10.7717/peerj.21671/supp-2 Supplemental Information 2 qPCR raw data in 40 microgram ox-LDL treatment vs. 25 microgram ox-LDL treatment in HUVECs Each data indicates the values of threshold cycle (Ct or Cq) in 40 microgram ox-LDL treatment vs. 25 microgram ox-LDL treatment in HUVECs

10.7717/peerj.21671/supp-3 Supplemental Information 3 MIQE checklist

## Additional Information and Declarations

### Data Availability

The following information was supplied regarding data availability: The raw data are available in the Supplemental Files.

## References

[ref-1] Bayyurt B, Arslan S (2025). Highly up-regulation of fas ligand gene expression after increasing in oxidized low-density lipoprotein. Black Sea Journal of Engineering and Science.

[ref-2] Boatright KM, Salvesen GS (2003). Mechanisms of caspase activation. Current Opinion in Cell Biology.

[ref-3] Chen J, Mehta JL, Haider N, Zhang X, Narula J, Li D (2004). Role of caspases in Ox-LDL-induced apoptotic cascade in human coronary artery endothelial cells. Circulation Research.

[ref-4] Cristaldi M, Buscetta M, Cimino M, La Mensa A, Giuffré MR, Fiore L, Carcione C, Bucchieri F, Rappa F, Coronnello C, Sciaraffa N, Amato S, Aronica TS, Lo Iacono G, Bertani A, Pace E, Cipollina C (2023). Caspase-8 activation by cigarette smoke induces pro-inflammatory cell death of human macrophages exposed to lipopolysaccharide. Cell Death & Disease.

[ref-5] Folkersen L, Persson J, Ekstrand J, Agardh HE, Hansson GK, Gabrielsen A, Hedin U, Paulsson-Berne G (2012). Prediction of ischemic events on the basis of transcriptomic and genomic profiling in patients undergoing carotid endarterectomy. Molecular Medicine.

[ref-6] Fritsch M, Günther SD, Schwarzer R, Albert MC, Schorn F, Werthenbach JP, Schiffmann LM, Stair N, Stocks H, Seeger JM, Lamkanfi M, Krönke M, Pasparakis M, Kashkar H (2019). Caspase-8 is the molecular switch for apoptosis, necroptosis and pyroptosis. Nature.

[ref-7] Galluzzi L, Vitale I, Aaronson SA, Abrams JM, Adam D, Agostinis P, Alnemri ES, Altucci L, Amelio I, Andrews DW, Annicchiarico-Petruzzelli M, Antonov AV, Arama E, Baehrecke EH, Barlev NA, Bazan NG, Bernassola F, Bertrand MJM, Bianchi K, Blagosklonny MV, Blomgren K, Borner C, Boya P, Brenner C, Campanella M, Candi E, Carmona-Gutierrez D, Cecconi F, Chan FK, Chandel NS, Cheng EH, Chipuk JE, Cidlowski JA, Ciechanover A, Cohen GM, Conrad M, Cubillos-Ruiz JR, Czabotar PE, D’Angiolella V, Dawson TM, Dawson VL, De Laurenzi V, De Maria R, Debatin KM, DeBerardinis RJ, Deshmukh M, Di Daniele N, Di Virgilio F, Dixit VM, Dixon SJ, Duckett CS, Dynlacht BD, El-Deiry WS, Elrod JW, Fimia GM, Fulda S, García-Sáez AJ, Garg AD, Garrido C, Gavathiotis E, Golstein P, Gottlieb E, Green DR, Greene LA, Gronemeyer H, Gross A, Hajnoczky G, Hardwick JM, Harris IS, Hengartner MO, Hetz C, Ichijo H, Jäättelä M, Joseph B, Jost PJ, Juin PP, Kaiser WJ, Karin M, Kaufmann T, Kepp O, Kimchi A, Kitsis RN, Klionsky DJ, Knight RA, Kumar S, Lee SW, Lemasters JJ, Levine B, Linkermann A, Lipton SA, Lockshin RA, López-Otín C, Lowe SW, Luedde T, Lugli E, MacFarlane M, Madeo F, Malewicz M, Malorni W, Manic G, Marine JC, Martin SJ, Martinou JC, Medema JP, Mehlen P, Meier P, Melino S, Miao EA, Molkentin JD, Moll UM, Muñoz Pinedo C, Nagata S, Nuñez G, Oberst A, Oren M, Overholtzer M, Pagano M, Panaretakis T, Pasparakis M, Penninger JM, Pereira DM, Pervaiz S, Peter ME, Piacentini M, Pinton P, Prehn JHM, Puthalakath H, Rabinovich GA, Rehm M, Rizzuto R, Rodrigues CMP, Rubinsztein DC, Rudel T, Ryan KM, Sayan E, Scorrano L, Shao F, Shi Y, Silke J, Simon HU, Sistigu A, Stockwell BR, Strasser A, Szabadkai G, Tait SWG, Tang D, Tavernarakis N, Thorburn A, Tsujimoto Y, Turk B, Van den Berghe T, Vandenabeele P, Vander Heiden MG, Villunger A, Virgin HW, Vousden KH, Vucic D, Wagner EF, Walczak H, Wallach D, Wang Y, Wells JA, Wood W, Yuan J, Zakeri Z, Zhivotovsky B, Zitvogel L, Melino G, Kroemer G (2018). Molecular mechanisms of cell death: recommendations of the Nomenclature Committee on Cell Death. Cell Death and Differentiation.

[ref-8] Gao F, Zhao Y, Zhang B, Xiao C, Sun Z, Gao Y, Dou X (2024). Orientin alleviates ox-LDL-induced oxidative stress, inflammation and apoptosis in human vascular endothelial cells by regulating Sestrin 1 (SESN1)-mediated autophagy. Journal of Molecular Histology.

[ref-9] Gao X, Lv T, Li G, Tse G, Liu T (2022). Association between atherosclerosis-related cardiovascular disease and uveitis: a systematic review and meta-analysis. Diagnostics.

[ref-10] Goldblatt ZE, Cirka HA, Billiar KL (2021). Mechanical regulation of apoptosis in the cardiovascular system. Annals of Biomedical Engineering.

[ref-11] Gong R, Li XY, Chen HJ, Xu CC, Fang HY, Xiang J, Wu YQ (2019). Role of heat shock protein 22 in the protective effect of geranylgeranylacetone in response to oxidized-LDL. Drug Design, Development and Therapy.

[ref-12] Grego A, Fernandes C, Fonseca I, Dias-Neto M, Costa R, Leite-Moreira A, Oliveira SM, Trindade F, Nogueira-Ferreira R (2025). Endothelial dysfunction in cardiovascular diseases: mechanisms and *in vitro* models. Molecular and Cellular Biochemistry.

[ref-13] Gundapaneni KK, Shyamala N, Galimudi RK, Kupsal K, Gantala SR, Padala C, Gunda P, Tupurani MA, Puranam K, Sahu SK, Hanumanth SR (2017). Polymorphic variants of Caspase genes (8 & 3) in the risk prediction of Coronary Artery Disease. Gene.

[ref-14] Guo X, Guo Y, Wang Z, Cao B, Zheng C, Zeng Z, Wei Y (2022). Reducing the damage of Ox-LDL/LOX-1 pathway to vascular endothelial barrier can inhibit atherosclerosis. Oxidative Medicine and Cellular Longevity.

[ref-15] Guo J, Ning Y, Pan D, Wu S, Gao X, Wang C, Guo L, Gu Y (2024). Identification of potential hub genes and regulatory networks of smoking-related endothelial dysfunction in atherosclerosis using bioinformatics analysis. Technology and Health Care.

[ref-16] He W, Zhao L, Wang P, Ren M, Han Y (2025). MiR-125b-5p ameliorates ox-LDL-induced vascular endothelial cell dysfunction by negatively regulating TNFSF4/TLR4/NF-κB signaling. BMC Biotechnology.

[ref-17] Jiang J, Hiron TK, Chalisey A, Malhotra Y, Agbaedeng T, O’Callaghan CA (2025). Ox-LDL induces a non-inflammatory response enriched for coronary artery disease risk in human endothelial cells. Scientific Reports.

[ref-18] Kanavos T, Birbas E (2025). Cell-specific role of apoptosis in the process of atherosclerosis. Aging Advances.

[ref-19] Kang H, Yu H, Fan J, Cao G (2021). Rotigotine protects against oxidized low-density lipoprotein(ox-LDL)-induced damages in human umbilical vein endothelial cells(HUVECs). Bioengineered.

[ref-20] Kearney CJ, Cullen SP, Tynan GA, Henry CM, Clancy D, Lavelle EC, Martin SJ (2015). Necroptosis suppresses inflammation *via* termination of TNF-or LPS-induced cytokine and chemokine production. Cell Death & Differentiation.

[ref-21] Kesavardhana S, Malireddi RKS, Kanneganti TD (2020). Caspases in cell death, inflammation, and pyroptosis. Annual Review of Immunology.

[ref-22] Lawal AO, Davids LM, Marnewick JL (2016). Diesel exhaust particles and endothelial cells dysfunction: an update. Toxicology in Vitro.

[ref-23] Li L, Chen Y, Shi C (2022). Nintedanib ameliorates oxidized low-density lipoprotein -induced inflammation and cellular senescence in vascular endothelial cells. Bioengineered.

[ref-24] Li D, Mehta JL (2000). Upregulation of endothelial receptor for oxidized LDL (LOX-1) by oxidized LDL and implications in apoptosis of human coronary artery endothelial cells: evidence from use of antisense LOX-1 mRNA and chemical inhibitors. Arteriosclerosis, Thrombosis, and Vascular Biology.

[ref-25] Liu Q, Liu Z, Zhou LJ, Cui YL, Xu JM (2020). The long noncoding RNA NKILA protects against myocardial ischaemic injury by enhancing myocardin expression *via* suppressing the NF-*κ*B signalling pathway. Experimental Cell Research.

[ref-26] Livak KJ, Schmittgen TD (2001). Analysis of relative gene expression data using real-time quantitative PCR and the 2(-Delta Delta C(T)) method. Methods.

[ref-27] Ma J, Ling J, Tong R, Guo J, Zhu Z (2024). siLOXL2 inhibits endothelial inflammatory response and endmt induced by ox-LDL. Cerebrovascular Diseases Extra.

[ref-28] Meng X, Zhang K, Xi Y, Wang Z, Zhu X, Zhang W (2026). Systematic identification and functional validation of CASP10 as a DNA-damage-responsive driver of endothelial pyroptosis in atherosclerosis. Journal of Cellular and Molecular Medicine.

[ref-29] Monteiro JP, Bennett M, Rodor J, Caudrillier A, Ulitsky I, Baker AH (2019). Endothelial function and dysfunction in the cardiovascular system: the long non-coding road. Cardiovascular Research.

[ref-30] Ni D, Lei C, Liu M, Peng J, Yi G, Mo Z (2024). Cell death in atherosclerosis. Cell Cycle.

[ref-31] Nishio E, Arimura S, Watanabe Y (1996). Oxidized LDL induces apoptosis in cultured smooth muscle cells: a possible role for 7-ketocholesterol. Biochemical and Biophysical Research Communications.

[ref-32] Oberst A, Dillon CP, Weinlich R, McCormick LL, Fitzgerald P, Pop C, Hakem R, Salvesen GS, Green DR (2011). Catalytic activity of the caspase-8-FLIP(L) complex inhibits RIPK3-dependent necrosis. Nature.

[ref-33] Ohsawa S, Vaughen J, Igaki T (2018). Cell extrusion: a stress-responsive force for good or evil in epithelial homeostasis. Developmental Cell.

[ref-34] Orning P, Lien E (2021). Multiple roles of caspase-8 in cell death, inflammation, and innate immunity. Journal of Leukocyte Biology.

[ref-35] Pavić J, Z˘ivanović M, Tanasković I, Pavić O, Stanković V, Virijević K, T Mladenović, Košarić J, Milićević B, Qamar SUR, Velicki L, Novaković I, Preveden A, Popović D, Tesić M, Seman S, Filipović N (2024). A machine learning approach to gene expression in hypertrophic cardiomyopathy. Pharmaceuticals.

[ref-36] Su Q, Sun Y, Ye Z, Yang H, Kong B, Li L (2018). Pinocembrin protects endothelial cells from oxidized LDL-induced injury. Cytokine.

[ref-37] Tian D, Liu L, Zeng GG, He J, Liu H, Ma D, Yang Z, Ma X, Cao Y, Xu C (2026). Caspase recruitment domain family member 8: a favorable target in the pathogenesis of atherosclerosis. Reviews in Cardiovascular Medicine.

[ref-38] Wang Q, Wang T, Liang S, Zhou L (2023). Ox-LDL-induced vascular smooth muscle cell dysfunction partly depends on the Circ_0044073/miR-377-3p/AURKA Axis in atherosclerosis. International Heart Journal.

[ref-39] Wang Z, Yang Y, Wang Q, Wang L, Zhao Y, Qian X, Feng R, Qian J (2026a). Pathological mechanisms and clinical research progress of endothelial dysfunction. Frontiers in Cardiovascular Medicine.

[ref-40] Wang S, Zhao B, Cui Y, Gui L, Fan J, Huang L (2026b). Role and mechanism of miR-222-5p in endothelial cell apoptosis. Molecular Medicine Reports.

[ref-41] Wu CY, Tang ZH, Jiang L, Li XF, Jiang ZS, Liu LS (2012). PCSK9 siRNA inhibits HUVEC apoptosis induced by ox-LDL *via* Bcl/Bax-caspase9-caspase3 pathway. Molecular and Cellular Biochemistry.

[ref-42] Zhang M, Jiang L (2016). Oxidized low-density lipoprotein decreases VEGFR2 expression in HUVECs and impairs angiogenesis. Experimental and Therapeutic Medicine.

[ref-43] Zhang W, Zhu C, Liao Y, Zhou M, Xu W, Zou Z (2024). Caspase-8 in inflammatory diseases: a potential therapeutic target. Cellular & Molecular Biology Letters.

[ref-44] Zhao Y, Malik S, Criqui MH, Allison MA, Budoff MJ, Sandfort V, Wong ND (2022). Coronary calcium density in relation to coronary heart disease and cardiovascular disease in adults with diabetes or metabolic syndrome: the Multi-ethnic Study of Atherosclerosis (MESA). BMC Cardiovascular Disorders.

[ref-45] Zhong B, Liu M, Bai C, Ruan Y, Wang Y, Qiu L, Hong Y, Wang X, Li L, Li B (2020). Caspase-8 induces lysosome-associated cell death in cancer cells. Molecular Therapy.

